# Nonsteroidal Anti‐Inflammatory Drugs and the Risk of Periodontitis Among Adults With Osteoarthritis: A Target Trial Emulation

**DOI:** 10.1111/jcpe.70145

**Published:** 2026-05-23

**Authors:** Ignacio Leiva‐Escobar, Aiswarya Puzhakkara Chennas, Zoheir Alayash, Stefan Lars Reckelkamm, Birte Holtfreter, Thomas Kocher, Sebastian‐Edgar Baumeister, Michael Nolde

**Affiliations:** ^1^ Institute of Health Services Research in Dentistry University of Münster Münster Germany; ^2^ Clinic for Periodontology and Conservative Dentistry University of Münster Münster Germany; ^3^ Department of Restorative Dentistry, Periodontology and Endodontology University Medicine Greifswald Greifswald Germany

**Keywords:** anti‐inflammatory agents, causality, nonsteroidal, observational studies as topic, periodontitis, therapeutic use

## Abstract

**Background:**

Nonsteroidal anti‐inflammatory drugs (NSAIDs) may mitigate periodontal tissue breakdown through cyclooxygenase (COX) inhibition; however, their long‐term effects on the risk of periodontitis remain unclear.

**Methods:**

We emulated target trials using US electronic health records (1995–2019) to compare the 5‐year risk of incident periodontitis among adults with osteoarthritis‐initiating NSAIDs versus acetaminophen. Eligible individuals had osteoarthritis, no prior periodontitis and no recent use of either medication. We estimated the risk of periodontitis using a pooled logistic regression model, adjusting for confounding via inverse probability weighting. Additionally, we conducted a subgroup analysis by NSAID COX selectivity, along with a negative control outcome analysis examining dental caries.

**Results:**

The 5‐year risk difference (NSAIDs – acetaminophen) was 0.13% (95% CI: −0.32%, 0.58%). Similar findings were observed across subgroups compared with acetaminophen: non‐selective NSAIDs, 0.11% (95% CI: −0.36%, 0.59%); preferential COX‐2 NSAIDs, 0.10% (95% CI: −0.71%, 0.92%). The 5‐year risk difference for dental caries was −0.27% (95% CI: −1.01%, 0.50%).

**Conclusion:**

We found no evidence that initiating NSAIDs, compared with acetaminophen, influenced the 5‐year risk of periodontitis, and no differences by COX selectivity. Our findings align with inconclusive evidence from smaller studies on NSAIDs as adjuncts in periodontitis treatment.

## 1 Introduction

Periodontitis is a highly prevalent chronic inflammatory disease characterised by progressive loss of periodontal ligament and alveolar bone support, leading to tooth loss and resulting in masticatory dysfunction, compromised nutrition and reduced quality of life (Hajishengallis and Korostoff 2017; Uy et al. 2022). In 2021, severe periodontitis remained one of the most common oral conditions globally, with an age‐standardised prevalence of approximately 12.5% (Bernabe et al. 2025). Beyond local tissue destruction, periodontitis has been linked to systemic conditions including cardiovascular, respiratory, endocrine and musculoskeletal diseases, underscoring its clinical importance and the need for effective strategies to prevent its onset and progression (Hajishengallis 2022; Herrera et al. 2023).

Periodontitis develops when a dysbiotic subgingival biofilm induces a sustained and dysregulated host inflammatory response. Although bacteria initiate the disease, tissue breakdown is largely driven by an exacerbated inflammatory cascade involving cytokines, matrix metalloproteinases (MMPs) and prostaglandins, particularly prostaglandin E_2_ (PGE_2_) (Hajishengallis and Korostoff 2017). This inflammatory basis has motivated interest in pharmacological agents that can modulate host response pathways involved in periodontal destruction.

Several interventional studies have investigated whether nonsteroidal anti‐inflammatory drugs (NSAIDs) can attenuate the progression of periodontitis. By inhibiting cyclooxygenase (COX) enzymes, NSAIDs reduce the synthesis of PGE_2_, a key mediator that up‐regulates MMPs and promotes osteoclast activity (Ren et al. 2023). Overall, findings from these studies are inconsistent and appear to depend on factors such as the specific NSAID used, the duration of treatment and whether the NSAID is administered alongside mechanical instrumentation (i.e., scaling and root planning [SRP]). Some trials reported a reduction in alveolar bone loss (Azoubel et al. 2008; Heasman et al. 1993; Jeffcoat et al. 1988), whereas others reported improvements in probing depth (PD) and clinical attachment loss (CAL) with prolonged adjunctive regimens (Aras et al. 2007; Farahmand et al. 2019; Yen et al. 2008). Short‐term NSAID regimens (7–10 days) consistently lower local inflammatory mediators (e.g., PGE_2_, MMP‐8), but these reductions rarely translate into clinical benefit (Kurtiş et al. 2007; Ozgören et al. 2014; Vardar et al. 2003). Notably, two trials evaluated NSAIDs without simultaneous instrumentation. One trial found that flurbiprofen 50 mg twice daily reduced alveolar bone loss (Jeffcoat et al. 1988), while the other reported that cyclic diclofenac potassium 50 mg twice daily for 10 days every 3 months improved PD and CAL during maintenance therapy (Oduncuoglu et al. 2018).

The use of NSAIDs for managing musculoskeletal pain is common (Machado et al. 2021). Observational studies reported that approximately 33%–65% of individuals with osteoarthritis (OA) in the United States receive prescribed NSAIDs for chronic pain management (Gore et al. 2012; Ide et al. 2024). Clinical guidelines for OA recommend NSAIDs as the primary analgesic option for chronic pain, whereas acetaminophen is conditionally recommended for patients who cannot tolerate NSAIDs or have contraindications (Kolasinski et al. 2020). Unlike NSAIDs, acetaminophen provides analgesia with minimal anti‐inflammatory activity because it weakly inhibits peripheral COX enzymes (Ohashi and Kohno 2020).

Given the current evidence and the plausible mechanistic pathway, we sought to answer the causal question: ‘Does the initiation of NSAIDs decrease the 5‐year risk of periodontitis compared to acetaminophen among adults with osteoarthritis?’ To address this question, we emulated a hypothetical target trial using US electronic health records (EHRs). We followed the principles of the target trial emulation (TTE) framework, which provides a structured approach for designing observational studies that mimic randomised trials, aiming to minimise self‐inflicted biases and strengthen causal interpretation of findings (Hernán 2021; Hernán and Robins 2016). We further evaluated whether the association differed between initiators of preferential COX‐2 inhibitors and non‐selective NSAIDs.

## 2 Materials and Methods

### 2.1 Specification of the (Hypothetical) Target Trial

We designed this observational analysis to emulate a hypothetical pragmatic trial—the target trial—that would have answered the causal question of interest. Because long‐term treatment with non‐aspirin NSAIDs is a common approach for symptom management among individuals with osteoarthritis, we restricted our analysis to this population to increase the likelihood of identifying long‐term users of the medication. Table 1 summarises the key components of the target trial specification that were emulated using EHRs.

**TABLE 1:** Target trial specification and its emulation using the All of Us Research Program.

Protocol component	Target trial specification	Target trial emulation
Eligibility criteria	Age ≥ 18 years between 1 January 1995 and 31 December 2019	Same as the target trial. In addition, individuals were required to have at least 1 year of health record history prior to baseline
	Diagnosed with osteoarthritis	Lookback: Infinite
	No history of prior periodontitis	Lookback: Infinite
	No NSAIDs or acetaminophen contraindication: Recent peptic ulcer, gastrointestinal bleeding, stroke, acute myocardial infarction. History of chronic kidney disease, heart failure, and severe liver disease	Lookback: 6 months Lookback: Infinite
	No edentulism	Lookback: Infinite
	No prior use of NSAIDs or acetaminophen	Lookback: 1 year
Treatment strategies	Individuals randomly assigned to Initiate NSAIDs, except for aspirin Initiate acetaminophen	Same as the trial, with initiation being defined as the first prescription during the trial year. For instance, for the 1995 trial, the first prescription was between 1 January 1995 and 31 December 1995
Assignment procedure	Eligible individuals were randomly assigned to one of the strategies. Individuals were aware of the strategy they are assigned	We assumed that individuals were randomly assigned within levels of their baseline covariates (see Table 2). Randomisation was emulated by using the stabilized inverse probability of treatment weighting (SIPTW)
Follow‐up	For each individual, follow‐up starts at the time of assignment to a strategy and ends at incident periodontitis, death, loss to follow‐up, 5 years or end of the study (31 December 2019), whichever comes first	Same as the trial. The end of the study was 31 December 2019
Outcome	Incident periodontitis	Same as the trial
Causal contrast	Intention‐to‐treat effect	Observational analogue of the intention to treat
Data analysis plan	Estimate the cumulative risk of periodontitis under each strategy and the risk differences with 95% confidence intervals	Same as the target trial for the intention to treat. Additionally, to analytically mimic randomisation, the SIPTW was applied

Abbreviation: NSAID, nonsteroidal anti‐inflammatory drug.

**FIGURE 1:**
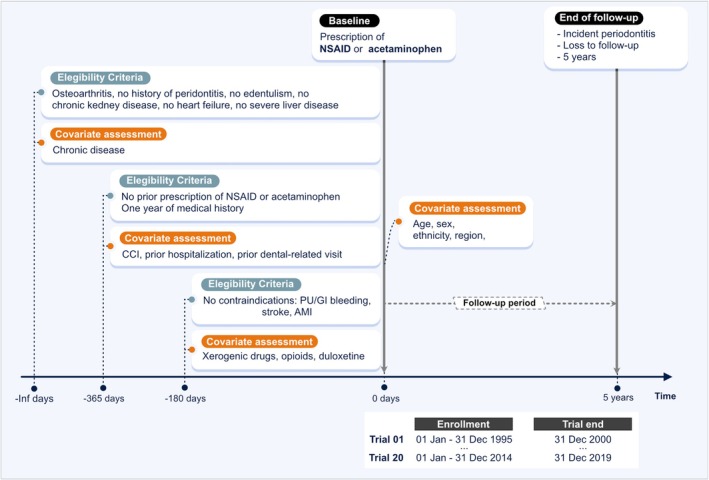
Assessment of eligibility criteria and covariates for each trial. The 1995 Trial enrolled people from 1 January until 31 December 1995, and concluded on 31 December 2000. AMI, acute myocardial infarction; CCI, Charlson comorbidity index; GI, gastrointestinal; NSAID, nonsteroidal anti‐inflammatory drug; PU, peptic ulcer.

Our hypothetical trial included individuals aged 18 or older with a diagnosis of osteoarthritis at cohort entry, no history of periodontitis, no edentulism, no contraindication to NSAIDs or acetaminophen (i.e., recent peptic ulcer, gastrointestinal bleeding, stroke, acute myocardial infarction, history of chronic kidney disease, heart failure and severe liver disease) and no prescription for NSAIDs or acetaminophen the previous year (Figure 1).

The treatment strategies to be compared were (1) initiation of NSAIDs versus (2) initiation of acetaminophen at baseline. Co‐prescriptions of opioids were allowed.

The outcome of interest was incident periodontitis, with follow‐up starting at treatment assignment (baseline) and concluding at the earliest occurrence of periodontitis, death, loss to follow‐up, 5 years of follow‐up or the end of the study period (31 December 2019).

The causal contrast of interest was the intention‐to‐treat effect of being assigned to one of the strategies. The analysis plan aimed to estimate the cumulative risk under each strategy and the risk differences using a pooled logistic regression model for the monthly probability of the outcome. Models were used to estimate the 5‐year risk of developing periodontitis.

This target trial specification allowed proper alignment of time zero (i.e., baseline), defined as the time at which individuals meet the eligibility criteria, are assigned to a treatment strategy and begin follow‐up. This alignment helped avoid self‐inflicted biases in observational analyses, including immortal time bias (Hernán et al. 2016; Leiva‐Escobar et al. 2026).

### 2.2 Emulation of the Target Trial

We emulated the hypothetical trial using data from the ‘All of Us Research Program’ (AoU RP), which provides longitudinal individual‐level patient data from healthcare centres across the United States, including EHRs, demographic information, physical measurements, questionnaires, biospecimens with genomic data and information from wearables/devices, from over 868,000 participants (All of Us Research Hub 2025). The EHR data is harmonised in accordance with the Observational Medical Outcomes Partnership (OMOP) Common Data Model. Source vocabularies are mapped to OMOP standard concepts identifiers, and medications are standardised using RxNorm identifiers (Denny et al. 2019; Ramirez et al. 2022).

We used the harmonised EHRs and questionnaire data to emulate each component of the target trial as closely as possible using predefined code lists (Table 1 and Section S1).

Following a common approach to increase statistical power, we emulated 20 sequential target trials, allowing individuals to contribute to multiple trials if they met the eligibility criteria more than once during the study period (Danaei et al. 2013; Hernán et al. 2008). Trials were initiated annually between 1995 and 2014, with a 1‐year enrolment period.

#### 2.2.1 Eligibility Criteria and Treatment Strategies

We structured each emulation by its corresponding ‘trial year’, defined as the 12‐month period during which eligibility and treatment initiation were assessed (e.g., 1995 trial year: 1 January –31 December 1995). For each trial year from 1995 to 2014, we identified individuals with osteoarthritis who had prescription records consistent with either the NSAID or acetaminophen strategy. The date of the first prescription in that year was set as the baseline. All remaining eligibility criteria were evaluated retrospectively from this baseline date using predefined code lists and lookback periods (Figure 1). The final cohort consisted of individuals who met all eligibility criteria at baseline.

#### 2.2.2 Outcome and Follow‐Up

We identified periodontitis diagnoses using OMOP standard concept identifiers (see Section S1). To ensure that only incident cases were included, we excluded individuals with any periodontitis diagnosis before baseline and considered diagnoses occurring thereafter as incident events. Individuals were followed from baseline until the first occurrence of periodontitis, death, loss to follow‐up (defined as a 180‐day gap without healthcare activity), 5 years of follow‐up or 31 December2019 (end of study), whichever occurred first.

#### 2.2.3 Covariates

We selected baseline characteristics based on subject‐matter knowledge to adjust for potential confounding (Table 2 and Sections S1 and S2). We used pre‐specified code lists to retrieve demographic and clinical data recorded on or before baseline (Figure 1). Additionally, we applied an algorithm to derive smoking status using information from both EHRs and questionnaires (see Section S3).

**TABLE 2:** Characteristics of participants with osteoarthritis across target trial emulations using the All of Us Research Program.

Characteristic	Before weighting			After weighting		
	acetaminophen (*n* = 10,085)	NSAIDs (*n* = 10,700)	ASMD	acetaminophen (*n* = 10143.9)	NSAIDs (*n* = 10425.2)	ASMD
Age (years)	57.5 (11.3)	55.9 (11.0)	0.144	56.6 (11.3)	56.3 (11.3)	0.023
Sex	0.029					0.008
Female	6294 (62.4)	6726 (62.9)		6481.8 (63.9)	6699.2 (64.3)	
Male	3632 (36.0)	3841 (35.9)		3519.1 (34.7)	3585.1 (34.4)	
Other	159 (1.6)	133 (1.2)		143.0 (1.4)	140.9 (1.4)	
Ethnicity			0.125			0.015
Hispanic or Latino	851 (8.4)	1303 (12.2)		1077.9 (10.6)	1156.4 (11.1)	
Not Hispanic or Latino	8862 (87.9)	9059 (84.7)		8725.4 (86.0)	8925.3 (85.6)	
Skip, prefer not to answer, or none of these	372 (3.7)	338 (3.2)		340.6 (3.4)	343.5 (3.3)	
Region			0.175			0.043
Far West	456 (4.5)	503 (4.7)		448.0 (4.4)	442.2 (4.2)	
Great Lakes	3061 (30.4)	3088 (28.9)		2943.3 (29.0)	2992.8 (28.7)	
Mideast	2421 (24.0)	1950 (18.2)		2088.5 (20.6)	2020.1 (19.4)	
New England	2535 (25.1)	3282 (30.7)		3123.7 (30.8)	3356.4 (32.2)	
Other areas^a^	< 20 (< 0.2)	21 (0.2)		< 20.0 (< 0.2)	< 20.0 (< 0.2)	
Plains	532 (5.3)	597 (5.6)		508.6 (5.0)	511.2 (4.9)	
Rocky Mountain^a^	> 20 (> 0.2)	85 (0.8)		> 20.0 (> 0.20)	> 20.0 (> 0.2)	
Southeast	726 (7.2)	895 (8.4)		707.4 (7.0)	775.1 (7.4)	
Southwest	259 (2.6)	279 (2.6)		234.6 (2.3)	240.1 (2.3)	
Education			0.053			0.020
Less than high school	570 (5.65)	737 (6.9)		668.0 (6.6)	717.6 (6.9)	
High school graduate (includes GED)	1664 (16.5)	1745 (16.3)		1690.6 (16.7)	1780.3 (17.1)	
College higher	7663 (76.0)	8000 (74.8)		7588.8 (74.8)	7714.0 (74.0)	
Skip, prefer not to answer or none of these	188 (1.9)	218 (2.0)		196.5 (1.9)	213.3 (2.0)	
Time since diagnosis of osteoarthritis	61.6 (60.6)	58.58 (56.3)	0.052	61.3 (58.6)	61.0 (57.7)	0.005
Smoking status			0.050			0.008
Current smoker	1413 (14.0)	1454 (13.6)		1405.3 (13.9)	1470.1 (14.1)	
Ex‐smoker	3172 (31.5)	3193 (29.8)		3107.4 (30.6)	3171.0 (30.4)	
Never smoker	5116 (50.7)	5685 (53.1)		5258.9 (51.8)	5403.1 (51.8)	
Unknown	384 (3.8)	368 (3.4)		372.3 (3.7)	381.0 (3.7)	
Charlson comorbidity index			0.133			0.013
0	6910 (68.5)	7957 (74.4)		7137.0 (70.4)	7321.7 (70.2)	
1	2260 (22.4)	2009 (18.8)		2172.5 (21.4)	2213.3 (21.2)	
2	533 (5.3)	445 (4.2)		491.5 (4.8)	516.3 (5.0)	
≥ 3	382 (3.8)	289 (2.7)		342.8 (3.4)	373.9 (3.6)	
Opioid use in the past 6 months	6335 (62.8)	1567 (14.6)	1.138	3828.0 (37.7)	3780.0 (36.3)	0.031
Prior dental‐related visit	414 (4.1)	382.00 (3.6)	0.028	397.9 (3.9)	421.1 (4.0)	0.006
Prior hospitalisation	1604 (15.9)	939.00 (8.8)	0.218	1290.7 (12.7)	1357.3 (13.0)	0.009
Xerogenic medication	4813 (47.7)	4013 (37.5)	0.208	4528.7 (44.6)	4795.5 (46.0)	0.027
Duloxetine	191 (1.9)	163 (1.5)	0.029	175.3 (1.7)	182.2 (1.7)	0.001
Rheumatoid arthritis	925 (9.2)	1014 (9.5)	0.010	997.8 (9.8)	1035.7 (9.9)	0.003
Gout	897 (8.9)	1131 (10.6)	0.057	998.1 (9.8)	1054.3 (10.1)	0.009
Hypertension	6183 (61.3)	6087 (56.9)	0.090	6031.5 (59.5)	6170.5 (59.2)	0.006
Diabetes mellitus	2347 (23.3)	2137 (19.8)	0.080	2214.0 (21.8)	2275.7 (21.8)	< 0.001
Chronic obstructive pulmonary disease	1067 (10.6)	871 (8.1)	0.084	970.6 (9.6)	999.9 (9.6)	0.001
Coronary heart disease	1704 (16.9)	1478 (13.8)	0.086	1570.7 (15.5)	1603.6 (15.4)	0.003

*Note:* Data are presented as mean (standard deviation) or number (percentage).

Abbreviations: ASMD, absolute standardized mean difference; GED, general educational development; NSAID, nonsteroidal anti‐inflammatory drug.

^a^ Cells with fewer than 20 participants were replaced by < 20, in accordance with the All of Us Research Program and Statistics Dissemination Policy.

#### 2.2.4 Causal Contrast

We report the observational analogue of the intention‐to‐treat effect, contrasting the causal effect of initiating the NSAID versus the acetaminophen strategy, regardless of subsequent adherence (Hernán and Hernández‐Díaz 2012; Murray et al. 2021).

#### 2.2.5 Statistical Analysis

We used stabilised inverse probability of treatment weights (SIPTW) to adjust for confounding and achieve conditional exchangeability at baseline (Chesnaye et al. 2022; Xu et al. 2010). The weights were estimated using the marginal and conditional probability of initiating NSAIDs at baseline (i.e., the propensity score). We estimated the propensity score by fitting a logistic regression model that included the baseline covariates listed in Table 2. Age and time since osteoarthritis were modelled using spline functions to allow for flexible, non‐linear relationships. We also included the ‘trial year’ indicator as a covariate to capture changes in prescribing preferences over time.

Covariate balance was assessed after weighting using the absolute standardised mean differences (ASMD), considering values < 0.1 to indicate adequate balance (Kainz et al. 2017). In addition, we visually inspected the overlap of the weighted PS between the treatment groups (see Section S7.1).

We then estimated cumulative risks under each treatment strategy and the corresponding risk differences by fitting two separate weighted pooled logistic regression models, one for each strategy, implemented via estimating equations (M‐estimation) (Zivich et al. 2025). This approach models the month‐specific hazard of periodontitis over the follow‐up period, allowing the underlying baseline risk to vary over time. Marginal cumulative risks under each treatment strategy and risk differences with 95% confidence intervals were obtained using the empirical (cluster‐robust) sandwich variance estimator.

All analyses were conducted as complete‐case analyses under the assumption that data were missing at random. Statistical analyses were performed in Python v. 3.10.16.

### 2.3 Subgroup Analyses

To explore whether the association between NSAID initiation and periodontitis differed by COX isoform affinity, we conducted a secondary analysis classifying NSAIDs into two groups: (1) NSAIDs with preferential COX‐2 inhibition (hereafter, preferential COX‐2 NSAIDs), including both selective COX‐2 inhibitors (coxibs) and NSAIDs with relatively high affinity for COX‐2 (meloxicam and etodolac); and (2) *non‐selective NSAIDs* (see Section S5.1). This classification reflects clinically relevant differences in safety profiles, particularly regarding gastrointestinal and cardiovascular adverse effects (Van Der Linden et al. 2009). Although both classes inhibit the inducible COX‐2 isoform, preferential COX‐2 inhibitors show relatively less COX‐1 inhibition, which may lead to differences in the estimated association.

We further evaluated the association between NSAIDs initiation and periodontitis by examining effect modification by sex, ethnicity and education level.

### 2.4 Negative Control Outcomes

Negative control outcome (NCO) analyses are proposed as a tool to assess potential bias from unmeasured or mismeasured confounders (Arnold and Ercumen 2016; Lipsitch et al. 2010; Núñez and Matthews 2025). We selected dental caries as the NCO because it is not expected to be causally affected by NSAIDs use, yet may share unmeasured oral health‐seeking behaviour.

This study adhered to the Transparent Reporting of Observational Studies Emulating a Target Trial (TARGET) guideline (Cashin et al. 2025).

### 2.5 Ethics Statement

This study complied with the All of Us Data User Code of Conduct and Data and Statistics Dissemination Policy. Only authorised authors who completed the All of Us Responsible Conduct of Research training accessed the data. Research conducted in the Controlled Tier is overseen by the All of Us Institutional Review Board and is designated as non–human subjects research under a data passport model (Protocol 2021‐02‐TN‐001). Therefore, IRB approval was not required for this study.

## 3 Results

Table 2 presents the baseline characteristics of the 20,785 eligible individuals across 20 sequential trials. Before weighting, NSAID initiators had more favourable Charlson Comorbidity Index (CCI) scores, fewer prior hospitalisations, differences in the region of residence and a higher proportion of Hispanic or Latino individuals, compared with the acetaminophen initiators at baseline. They also had fewer xerogenic medications and opioid prescriptions. After weighting, all baseline covariates were well balanced, with ASMD below 0.1 and a good overlapping of the propensity score distribution (Figure S6).

**FIGURE 2:**
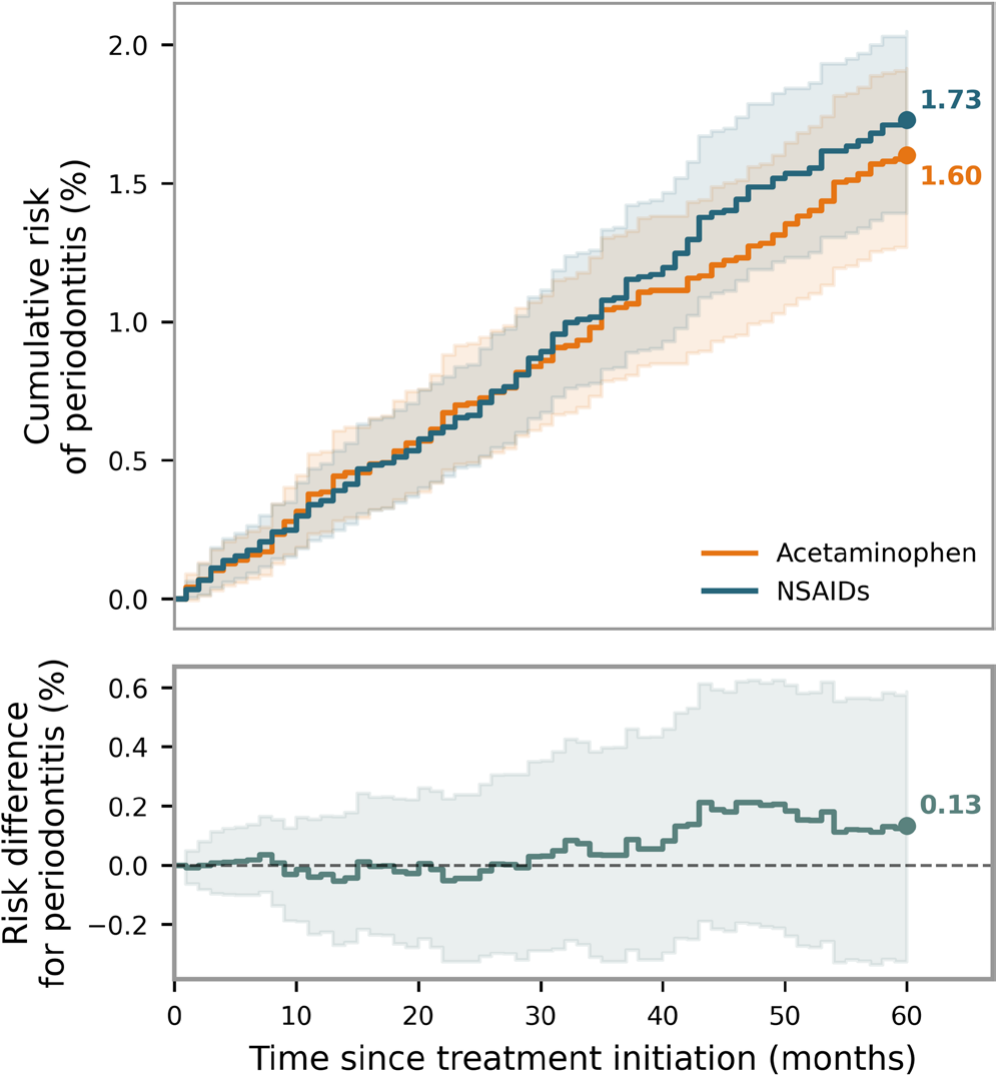
Cumulative risk and risk difference among individuals with osteoarthritis initiating either NSAID or acetaminophen. The top panel shows the cumulative risk for patients initiating NSAIDs (blue line) or acetaminophen initiators (orange line). The bottom panel shows the estimated risk differences, with the grey dashed line indicating the null effect. Shaded areas represent 95% confidence intervals.

Across the emulated trials, 294 individuals developed periodontitis during the 5‐year follow‐up (median 5 years, interquartile range 3.6–5 years). Figure 2 shows the adjusted cumulative incidence curves for periodontitis among NSAID and acetaminophen initiators. At year 5, the estimated risk was 1.73% for NSAID initiators and 1.60% for acetaminophen initiators, yielding an observational analogue of the intention‐to‐treat 5‐year risk difference for periodontitis of 0.13% (95% CI: −0.32%, 0.58%).

**FIGURE 3:**
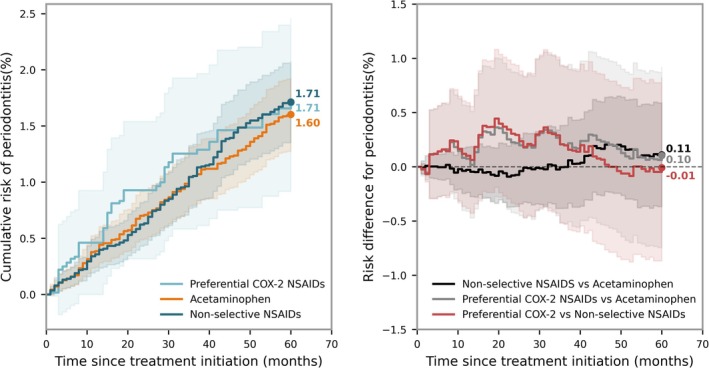
Cumulative risk and risk difference among individuals with osteoarthritis initiating (1) preferential COX‐2 NSAIDs (light blue), (2) non‐selective NSAIDs (blue) or (3) acetaminophen (orange). The left panel shows the cumulative risk of periodontitis for patients initiating each treatment. The right panel shows the estimated risk differences, with the grey dashed line indicating the null effect. Shaded areas represent 95% confidence intervals.

Among NSAID users, 8548 initiated a non‐selective NSAID and 2152 initiated a preferential COX‐2 NSAID. Figure 3 shows the adjusted cumulative incidence estimates for these groups. By Year 5, the estimated risk of periodontitis was 1.71 for both non‐selective and preferential COX‐2 NSAID initiators. The 5‐year risk difference comparing non‐selective NSAID with acetaminophen initiators was 0.11% (95% CI: −0.36%, 0.59%). A similar 5‐year risk difference was observed for preferential COX‐2 NSAID versus acetaminophen initiators, 0.10% (95% CI: −0.71%, 0.92%). The risk difference comparing preferential COX‐2 with non‐selective NSAID initiators at 5 years was −0.01% (95% CI: −0.83%, 0.82%).

There was no evidence of effect modification by sex, ethnicity or educational attainment for the 5‐year risk difference (Figures S3–S5).

**FIGURE 4:**
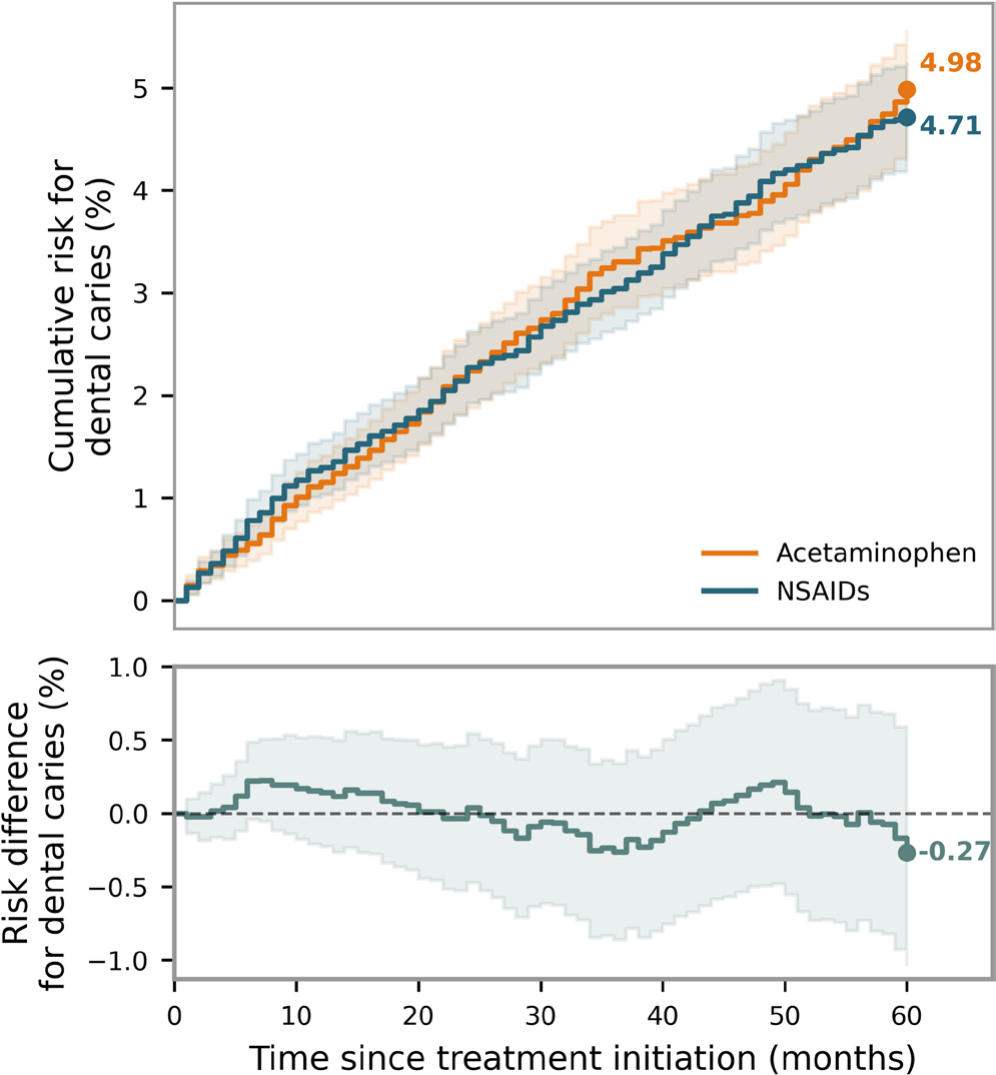
Cumulative risk and risk difference for dental caries, the negative control outcome, among individuals with osteoarthritis initiating either NSAID or acetaminophen. The top panel shows the cumulative risk for patients initiating NSAIDs (blue line) or acetaminophen initiators (orange line). The bottom panel shows the estimated risk differences, with the grey dashed line indicating the null effect. Shaded areas represent 95% confidence intervals.

As shown in Figure 4, the estimated 5‐year risk difference for the NCO, namely dental caries, was −0.27% (95% CI: −1.01%, 0.50%).

## 4 Discussion

After emulating 20 sequential target trials using the EHRs of 20,785 individuals, we found no evidence of a difference in the 5‐year risk of incident periodontitis between NSAID—irrespective of the COX‐2 selectivity—and acetaminophen initiators. These findings align with the existing evidence suggesting that the therapeutic benefit of NSAIDs in periodontal treatment remains inconsistent (Donos et al. 2020; Sanz et al. 2020).

To our knowledge, no previous studies have evaluated NSAID use in relation to incident periodontitis. Existing evidence is limited to interventional trials in patients with established disease, in which various NSAIDs (e.g., naproxen, diclofenac, etoricoxib and celecoxib) were evaluated in different dosing regimens, most often as adjuncts to SRP. However, trial findings are heterogeneous, with some studies reporting no significant clinical improvement in clinical parameters (Aras et al. 2007; Kurtiş et al. 2007; Ozgören et al. 2014) and others showing significant effects on inflammatory mediators and clinical response (Oduncuoglu et al. 2018; Vardar et al. 2003; Yen et al. 2008).

When examining whether the association differed by the type of NSAID initiated, we similarly did not observe meaningful differences in the 5‐year risk of periodontitis. This analysis was motivated by the distinct safety profiles of NSAID subclasses, particularly the gastrointestinal adverse effects associated with factors such as prolonged use, which may influence NSAID discontinuation. Although our observational analogue of the intention‐to‐treat analysis does not capture post‐initiation treatment changes, we hypothesised that if NSAIDs exert a measurable protective effect, initiators of preferential COX‐2 NSAIDs, who experience fewer COX‐1‐related adverse effects, may show a lower risk of developing periodontitis because these agents may be better tolerated and therefore used for longer periods. However, only about 20% of NSAID initiators received a preferential COX‐2 agent, and this group contributed to 31 periodontitis events. Thus, our subgroup findings may have been underpowered to detect any significant heterogeneity based on the NSAIDs' selectivity.

Our NCO result was close to the null, suggesting limited residual confounding from unmeasured oral health–seeking behaviours. This supports our assumption that NSAID and acetaminophen initiators were broadly comparable in their underlying patterns of dental care use. Although access to dental services could depend on insurance coverage and plausibly introduce unobserved differences in care‐seeking and in the outcome detection, the NCO findings substantially reduce this concern.

A strength of our study is that we used the target trial emulation framework, which, compared with conventional observational studies, minimises potential design‐related biases, including selection bias due to prevalent users and immortal time bias arising from assigning individuals based on postbaseline variables (Dickerman et al. 2019; Hernán et al. 2016). Combined with the application of SIPTW to adjust for a comprehensive set of confounders, this framework supports a causal interpretation of our findings.

Our study had some potential limitations. First, evaluating long‐term NSAID effects is challenging due to variation in indications (acute vs. chronic pain), intermittent adherence and discontinuations driven by adverse events (Salis and Sainsbury 2024). In addition, over‐the‐counter NSAID use may lead to exposure misclassification, as non‐prescription use is not captured in the EHR; such misclassification is expected to be non‐differential with respect to periodontitis onset and would likely attenuate the estimated association towards the null.

Second, as in any observational study, individuals were not randomised to a treatment strategy. Consequently, differences in baseline characteristics between NSAID and acetaminophen initiators could confound the results. To address this, we analytically emulated randomisation by adjusting for baseline covariates. Moreover, confounding by indication is expected to be limited when assessing unintended effects, as in our study (Miettinen 1983). We also performed an NCO analysis to evaluate the extent of residual confounding. Nonetheless, residual confounding may still persist because of unmeasured confounding factors such as oral hygiene and smoking intensity, or because the measured variables may not fully capture the underlying sources of confounding. Third, periodontitis diagnoses were ascertained from EHRs as a binary variable. In contrast, intervational studies typically assess periodontal status using detailed and standarised clinical measures, whereas routine EHR data may be less granular and incomplete. To date, oral health records in AoU RP have not yet been validated, and we were unable to determine the source of the recorded diagnoses. This limits the interpretation of the outcome, as the validity of the diagnosis may differ depending on who entered the record. While recorded diagnoses may reflect true clinical assessments made by dental professionals, they may also represent information transferred from dental documentation, clinical correspondence, patient self‐reports or administrative coding. This raises the possibility of non‐differential misclassification or under‐ascertainment of periodontitis, which would likely bias the findings towards the null. These issues underscore the need to improve the quality and detail of periodontal data recorded in healthcare databases (Mullins et al. 2021).

Finally, our estimates correspond to the effect of treatment initiation, regardless of post‐initiation treatment decisions, including discontinuation, switching or combination therapy, all of which may reduce the contrast between treatment groups over time. Accordingly, the absence of an observed effect should be interpreted with caution, as it may indicate either a true null effect or attenuation due to adherence patterns (Hernán and Hernández‐Díaz 2012). Estimating alternative causal effects via an analogous per‐protocol approach was precluded by data constraints, specifically the absence of exhaustive longitudinal exposure data required to model treatment adherence and satisfy the underlying assumptions inherent to a time‐varying framework (Hernán and Hernández‐Díaz 2012; Murray et al. 2021).

## 5 Conclusion

Our findings suggest that NSAIDs do not reduce the 5‐year incidence of periodontitis compared with acetaminophen, among individuals with osteoarthritis. The target trial emulation helped minimise biases that commonly affect observational studies. This analysis also highlights the value and feasibility of leveraging EHR data to evaluate treatment effects in dentistry and to generate meaningful evidence for clinical decision making.

## Author Contributions

Ignacio Leiva‐Escobar contributed to the conception, design, data acquisition, analysis and interpretation, and drafted and critically revised the manuscript. Sebastian‐Edgar Baumeister and Michael Nolde contributed to the conception, design, data acquisition and interpretation and drafted and critically revised the manuscript. Aiswarya Puzhakkara Chennas, Zoheir Alayash, Stefan Lars Reckelkamm, Birte Holtfreter, and Thomas Kocher contributed to the conception and design, and critically revised the manuscript. All authors gave final approval and agreed to be accountable for all aspects of the work.

## Funding

This study was supported by the Deutsche Forschungsgemeinschaft (DFG, grant number 552087827).

## Conflicts of Interest

The authors declare no conflicts of interest.

## Supporting information


**Data S1:** Code lists and definition of variables.
**Table S1:** Nonsteroidal anti‐inflammatory drug RxNorm codes.
**Table S2:** Comorbidity concept IDs.
**Table S3:** Xerogenic medication RxNorm codes.
**Section S2**. US state classification.
**Section S3**. Smoking status classification.
**Table S4:** Smoking status concept IDs.
**Figure S1:** Diagram of the smoking status classification workflow.
**Section S4**. Directed acyclic graph.
**Figure S2:** Directed acyclic graph.
**Section S5**. Subgroup analysis.
**Table S5:** Nonsteroidal anti‐inflammatory drug classification.
**Figure S3:** Cumulative risk of periodontitis and risk difference for periodontitis, by sex.
**Figure S4:** Cumulative risk of periodontitis and risk difference for periodontitis, by ethnicity.
**Figure S5:** Cumulative risk of periodontitis and risk difference for periodontitis, by education level.
**Section S6**. Negative control outcome.
**Section S7**. Additional results.
**Figure S6:** Weighted propensity score density among nonsteroidal anti‐inflammatory drug initiators and acetaminophen initiators.
**Figure S7:** Weighted propensity score density among COX‐2 preferential agents, nonselective COX inhibitors, and acetaminophen initiators.

## Data Availability

The data that support the findings of this study are available from All of Us Resaech Program. Restrictions apply to the availability of these data, which were used under license for this study. Data are available from https://allofus.nih.gov with the permission of All of Us Resaech Program.
