# Supplementary material for: Nonsteroidal Anti‐Inflammatory Drugs and the Risk of Periodontitis Among Adults With Osteoarthritis: A Target Trial Emulation
Source: J Clin Periodontol. 2026 May 23;53(8):1301–11. doi: 10.1111/jcpe.70145 (PMC13371416; doi:10.1111/jcpe.70145)
Supplement: Supplementary file 1 — Data S1: Code lists and definition of variables. Table S1: Nonsteroidal anti‐inflammatory drug RxNorm codes. Table S2: Comorbidity concept IDs. Table S3: Xerogenic medication RxNorm codes. Section S2. US state classification. Section S3. Smoking status classification. Table S4: Smoking status concept IDs. Figure S1: Diagram of the smoking status classification workflow. Section S4. Directed acyclic graph. Figure S2: Directed acyclic graph. Section S5. Subgroup analysis. Table S5: Nonsteroidal anti‐inflammatory drug classification. Figure S3: Cumulative risk of periodontitis and risk difference for periodontitis, by sex. Figure S4: Cumulative risk of periodontitis and risk difference for periodontitis, by ethnicity. Figure S5: Cumulative risk of periodontitis and risk difference for periodontitis, by education level. Section S6. Negative control outcome. Section S7. Additional results. Figure S6: Weighted propensity score density among nonsteroidal anti‐inflammatory drug initiators and acetaminophen initiators. Figure S7: Weighted propensity score density among COX‐2 preferential agents, nonselective COX inhibitors, and acetaminophen initiators. [file JCPE-53-1301-s001.docx]

**Supplementary material for**

**Nonsteroidal anti-inflammatory drugs and the risk of periodontitis**

Ignacio Leiva-Escobar^1^, Aiswarya Puzhakkara Chennas^1^, Zoheir Alayash^1^, Stefan Lars Reckelkamm^1,2,^ Birte Holtfreter^3^, Thomas Kocher^3^, Sebastian-Edgar Baumeister^1^, Michael Nolde^1^

^1^ Institute of Health Services Research in Dentistry, University of Münster, Münster, Germany

^2^ Clinic for Periodontology and Conservative Dentistry, University of Münster, Münster, Germany.

^3^ Department of Restorative Dentistry, Periodontology and Endodontology, University Medicine Greifswald, Greifswald, Germany.

**This file includes**

[1. Code lists 2](#_Toc219189345)

[1.1 Context 2](#_Toc219189346)

[1.2 Exposure: Nonsteroidal anti-inflammatory drugs and Acetaminophen 3](#_Toc219189347)

[1.3 Periodontitis 3](#_Toc219189348)

[1.4 Comorbidities 3](#_Toc219189349)

[1.5 Charlson comorbidity index 4](#_Toc219189350)

[1.6 Code list for xerogenic medications 5](#_Toc219189351)

[1.7 Opioids and duloxetine 5](#_Toc219189352)

[2. State classification 5](#_Toc219189353)

[3. Smoking Status 6](#_Toc219189354)

[3.1 Survey questions and code list 6](#_Toc219189355)

[3.2 Ascertainment process 7](#_Toc219189356)

[4. Directed acyclic graph 8](#_Toc219189357)

[5. Subgroup analysis 8](#_Toc219189358)

[5.1 NSAID affinity 8](#_Toc219189359)

[5.2 Sex, ethnicity, and education 10](#_Toc219189360)

[6. Negative control outcome 12](#_Toc219189361)

[7. Additional results 12](#_Toc219189362)

[7.1 Propensity score evaluation 12](#_Toc219189363)

# Code lists

## Context

This document describes the process used to generate the code list. The All of Us data are structured using the OMOP Common Data Model (CDM), in which source codes from multiple vocabularies (e.g., SNOMED CT, ICD-10, LOINC) are mapped to standard SNOMED CT concepts, each of which is assigned a unique OMOP concept ID. Concepts in OMOP are arranged in a parent–child hierarchy: selecting a higher-level (parent) concept retrieves all related descendant concepts. This approach is useful when a broad set of related conditions is needed. However, descendant lists may include non-specific or clinically irrelevant concepts or may omit important terms pertinent to the condition of interest. Therefore, it is essential to verify that the descendants of any chosen parent concept accurately capture the intended condition while excluding unrelated concepts.

To ensure accuracy, we compared the list of descendant concepts retrieved in OMOP with a published reference code list. The reference code list was used as the standard against which to evaluate completeness and specificity. Any relevant descendant missing from the reference list was added, and any descendant concept that did not represent the target condition was removed. This process ensured that the final code list comprehensively reflected the condition of interest without including extraneous terms.

## Exposure: Nonsteroidal anti-inflammatory drugs and Acetaminophen

**Table S1.** Ingredient names and their respective RxNorm code.

| **Medication** | **Ingredient (RxNorm concept ID)** |
| --- | --- |
| NSAIDs | **Included:** Aceclofenac (19029393); Acemetacin (19029398); Alclofenac (40798610); Alminoprofen (19018431); Benoxaprofen (40798646); Benzydamine (19019620); Bufexamac (19032724); Bumadizone (19039703); Celecoxib (1118084); Clofezone (19018512); Dexibuprofen (35891918); Dexketoprofen (19056874); Diacetylrhein (19088915); Diclofenac (1124300); Diflunisal (1126128); Difenpiramide (36860684); Droxicam (19056645); Etodolac (1195492); Etoricoxib (19011355); Fenbufen (19095703); Fenoprofen (1153928); Fentiazac (36849155); Feprazone (19135796); Flufenamic acid (19110711); Flunoxaprofen (36862145); Flurbiprofen (1156378); Ibuprofen (1177480); Ibuproxam (2100514); Indomethacin (1178663); Indoprofen (40798896); Kebuzone (19069191); Ketoprofen (1185922); Ketorolac (1136980); Lonazolac (19071933); Lornoxicam (19049709); Meclofenamic acid (19125097); Meloxicam (1150345); Mofebutazone (19072129); Morniflumate (19072152); Nabumetone (1113648); Naproxen (1115008); Naproxcinod (36862288); Niflumic acid (19019023); Nimesulide (19069425); Orgotein (19082874); Oxaceprol (19003691); Oxametacin (36854478); Oxaprozin (1118045); Oxyphenbutazone (19025925); Parecoxib (19003570); Pelubiprofen (43009024); Phenylbutazone (1135710); Piroxicam (1146810); Pirprofen (40799029); Polmacoxib (43009007); Polysulfated glycosaminoglycan (40234351); Proglumetacin (19029327); Proquazone (747079); Rofecoxib (1189754); Salsalate (1137460); Sulindac (1236607); Suprofen (1036636); Tenidap (36855233); Tenoxicam (19041220); Tolfenamic acid (19042155); Tolmetin (1102917); Valdecoxib (1103374); Zomepirac (19102108). **Excluded:** Aspirin (1112807) |
| Acetaminophen | Acetaminophen (1125315) |

## Periodontitis

Periodontitis was identified using OMOP concept ID 141608. We excluded individuals with any recorded diagnosis of periodontitis before baseline, and classified as incident cases those occurring after the baseline date

## Comorbidities

We used published code lists to identify the relevant concepts and reviewed each code to ensure its appropriateness for the intended condition. When applicable, additional codes were incorporated by examining the descendant concepts of selected parent OMOP terms

**Table S2.** Published code list used to retrieve comorbidities.

| **Condition** | **Parent OMOP**  **concept ID** | **Published code list** |
| --- | --- | --- |
| Osteoarthritis | 80180 | OpenCodelists Version ID [2beaec27](https://www.opencodelists.org/codelist/user/speed-vm/osteoarthritis-snomed-ct/2beaec27/) |
| Peptic Ulcer/GI Bleed | 4027663/192671 | OpenCodelists Version ID [4d790b4f](https://www.opencodelists.org/codelist/opensafely/gi-bleed-or-ulcer-snomed/2020-04-08/#full-list) |
| Acute myocardial infarction | 312327 | HDRUK Phenotype Library [PH1782 v.3721](https://phenotypes.healthdatagateway.org/phenotypes/PH1782/version/3721/detail/) &  [PH1796 v.3751](https://phenotypes.healthdatagateway.org/phenotypes/PH1796/version/3751/detail/) |
| Heart failure | 316139 | - HDRUK Phenotype Library [PH1782 v.3721](https://phenotypes.healthdatagateway.org/phenotypes/PH1782/version/3721/detail/) & [PH1749 v.3681](https://phenotypes.healthdatagateway.org/phenotypes/PH1749/version/3681/detail/) |
| Stroke and transient ischemic attack | 381316/373503 | - OpenCodelists Version ID [39012916](https://www.opencodelists.org/codelist/bristol/multimorbidity_stroketransient-ischemic-attack/39012916/#full-list) - HDRUK Phenotype Library [PH1773 v.3712](https://phenotypes.healthdatagateway.org/phenotypes/PH1773/version/3712/detail/) & [PH1723 v.3651](https://phenotypes.healthdatagateway.org/phenotypes/PH1723/version/3651/detail/) |
| Coronary heart disease | 317576 | - OpenCodelists Version ID [20250627](https://www.opencodelists.org/codelist/nhsd-primary-care-domain-refsets/chd_cod/20250627/) |
| Hypertension | 316866 | - HDRUK Phenotype Library [PH1752 v.3685](https://phenotypes.healthdatagateway.org/phenotypes/PH1752/version/3685/detail/) |
| Kidney disease | 198124 | - HDRUK Phenotype Library [PH950 v.2128](https://phenotypes.healthdatagateway.org/phenotypes/PH950/version/2128/detail/) |
| Liver disease | --- | - HDRUK Phenotype Library [PH1014 v.2192](https://phenotypes.healthdatagateway.org/phenotypes/PH1014/version/2192/detail/) |
| Rheumatoid arthritis | 80809 | - OpenCodelists Version ID [2dce1af8](https://www.opencodelists.org/codelist/nhsd-primary-care-domain-refsets/c19rarth_cod/20250627/#full-list) & [363b9726](https://www.opencodelists.org/codelist/nhsd-primary-care-domain-refsets/rarth_cod/20250627/#full-list) |
| Gout | 440674 | - OpenCodelists Version ID [133992d4](https://www.opencodelists.org/codelist/user/markdrussell/gout/133992d4/) |
| Diabetes | 201820 | - OpenCodelists Version ID [2ad8f0b0](https://www.opencodelists.org/codelist/bristol/multimorbidity_diabetes/2ad8f0b0/#full-list) |
| Chronic obstructive pulmonary disease | 255573 | - OpenCodelists Version ID [20250627](https://www.opencodelists.org/codelist/nhsd-primary-care-domain-refsets/copd_cod/20250627/) - HDRUK Phenotype Library [PH991 v.2169](https://phenotypes.healthdatagateway.org/phenotypes/PH991/version/2169/detail/) |
| Obesity | 433736 | - HDRUK Phenotype Library [PH989 v.2167](https://phenotypes.healthdatagateway.org/phenotypes/PH989/version/2167/detail/) |

## Charlson comorbidity index

We calculated the Charlson Comorbidity Index (CCI) using the adaptation proposed by Fortin et al. (2022), which is based on standardized SNOMED CT terminology. Each code was reviewed and mapped to its corresponding standard OMOP concept ID to ensure proper alignment with the OMOP CDM.

## Code list for xerogenic medications

**Table S3.** List of ingredients associated with xerogenic (dry mouth–inducing) effects.

| **Ingredient** | **RxNorm** | **Ingredient** | **RxNorm** | **Ingredient** | **RxNorm** |
| --- | --- | --- | --- | --- | --- |
| **Alendronate** | 1557272 | **Doxepin** | 738156 | **Oxybutynin** | 918906 |
| **Amisulpride** | 19057607 | **Duloxetine** | 715259 | **Paliperidone** | 703244 |
| **Amitriptyline** | 710062 | **Ebastine** | 19092433 | **Paroxetine** | 722031 |
| **Aripiprazole** | 757688 | **Escitalopram** | 715939 | **Perphenazine** | 733008 |
| **Asenapine** | 40164052 | **Etravirine** | 1758536 | **Pregabalin** | 734354 |
| **Baclofen** | 715233 | **Fentanyl** | 1154029 | **Quetiapine** | 766814 |
| **Brimonidine** | 915542 | **Fluoxetine** | 755695 | **Raltegravir** | 1712889 |
| **Buprenorphine** | 1133201 | **Gabapentin** | 797399 | **Reboxetine** | 19084693 |
| **Bupropion** | 750982 | **Haloperidol** | 766529 | **Risperidone** | 735979 |
| **Butorphanol** | 1133732 | **Imipramine** | 778268 | **Rotigotine** | 786426 |
| **Cetirizine** | 1149196 | **Lamivudine** | 1704183 | **Saquinavir** | 1746244 |
| **Chlorpromazine** | 794852 | **Levocetirizine** | 1136422 | **Sertindole** | 19050633 |
| **Citalopram** | 797617 | **Lisdexamfetamine** | 709567 | **Sertraline** | 739138 |
| **Clonidine** | 1398937 | **Lithium** | 19124477 | **Tapentadol** | 19026459 |
| **Clozapine** | 800878 | **Loxapine** | 792263 | **Tiotropium** | 1106776 |
| **Desipramine** | 716968 | **Maraviroc** | 1787101 | **Tizanidine** | 778474 |
| **Desloratadine** | 1103006 | **Methylphenidate** | 705944 | **Tramadol** | 1103314 |
| **Desvenlafaxine** | 717607 | **Morphine** | 1110410 | **Valproate** | 745466 |
| **Dexmethylphenidate** | 731533 | **Nevirapine** | 1769389 | **Venlafaxine** | 743670 |
| **Didanosine** | 1724869 | **Nicotine** | 718583 | **Vortioxetine** | 44507700 |
| **Dihydrocodeine** | 1189596 | **Nortriptyline** | 721724 | **Ziprasidone** | 712615 |
| **Dosulepin** | 19037989 | **Olanzapine** | 785788 |  |  |

## Opioids and duloxetine

***Medication name (RxNorm):***

Codeine (1201620); Dihydrocodeine (1189596); Hydrocodone (1174888); Hydromorphone (1126658); Morphine (1110410); Oxycodone (1124957); Propoxyphene (1153664); Tramadol (1103314), duloxetine (715259)

# State classification

We grouped U.S. states based on the Bureau of Economic Analysis (BEA) eight-region scheme, which divides the country into areas with similar geography and economic characteristics (New England, Mideast, Great Lakes, Plains, Southeast, Southwest, Rocky Mountain, Far West). This grouping helps us capture broad health and socioeconomic patterns across regions.

# Smoking Status

## Survey questions and code list

We applied an algorithm using information from survey data and electronic health records as follows.

**Survey data**

We selected the following questions:

1. Have you smoked at least 100 cigarettes in your entire life? (There are 20 cigarettes in a pack.)? ^1^

Response: Yes (has branching^1^), No, don’t know, prefer not to answer

If the answer is Yes, then

^1^ How old were you when you first started regular cigarette smoking?

Response: Indicate Age, don’t know, prefer not to answer

1. If you have completely stopped smoking cigarettes, about how old were you when you stopped?

Response: Indicate Age, don’t know, prefer not to answer

^1^Never smoker: An adult who has never smoked, or who has smoked less than 100 cigarettes in his or her lifetime.

**Table S4.** Code list for smoking status.

| **Status** | **Concept ID** | **Concept name** | **Domain** |
| --- | --- | --- | --- |
| Current smoker | 903652 | Findings of tobacco or its derivatives use or exposure | Observation |
|  | 903659 | Light cigarette smoker (1-9 cigarettes per day) | Observation |
|  | 903660 | Moderate cigarette smoker (10-19 cigarettes per day) | Observation |
|  | 903661 | Heavy cigarette smoker (20-39 cigarettes per day) | Observation |
|  | 903662 | Very heavy cigarette smoker (more or equal to 40 cigarettes per day) | Observation |
|  | 903657 | Cigarette smoker | Observation |
|  | 4026923 | Smokes in bed | Observation |
|  | 903658 | Trivial cigarette smoker (0-1 cigarettes per day) | Observation |
|  | 903650 | Cigarettes pack-years smoked during life | Measurement |
|  | 903664 | Cigar smoker | Observation |
|  | 903663 | Pipe smoker | Observation |
|  | 1246020 | Provision of written information about secondhand smoke exposure | Observation |
|  | 4010323 | Cigarette smoke | Observation |
|  | 4028286 | Smoker's respiratory syndrome | Condition |
|  | 4010813 | Tobacco smoke | Observation |
|  | 44799960 | Asthma trigger - tobacco smoke | Condition |
|  | 764469 | Episodic dependence on cigarette smoking | Condition |
| Ex-smoker | 903651 | Currently doesn't use tobacco or its derivatives | Observation |
|  | 4052465 | Ex-pipe smoker | Observation |
|  | 903652 | Findings of tobacco or its derivatives use or exposure | Observation |
|  | 1077376 | Stopped smoking in first trimester | Observation |
|  | 1077377 | Stopped smoking after first trimester | Observation |
|  | 4090846 | Stop smoking monitor default | Observation |
| Never smoker | 42872410 | Never smoked any substance | Observation |
|  | 903653 | Never used tobacco or its derivatives | Observation |

## Ascertainment process

We determined smoking status using both survey data and electronic health records (EHR). Smoking status was assessed at or before the baseline date. For survey data, we used participants’ reported ages for key milestones (e.g., age at smoking initiation and cessation). For EHR data, we included only smoking-related records dated on or before the index date. Because smoking status is often under-recorded or inconsistently documented in EHRs (Patel et al., 2020; Polubriaginof et al., 2017), EHR-derived information was used solely to supplement survey data when the latter were incomplete or missing.


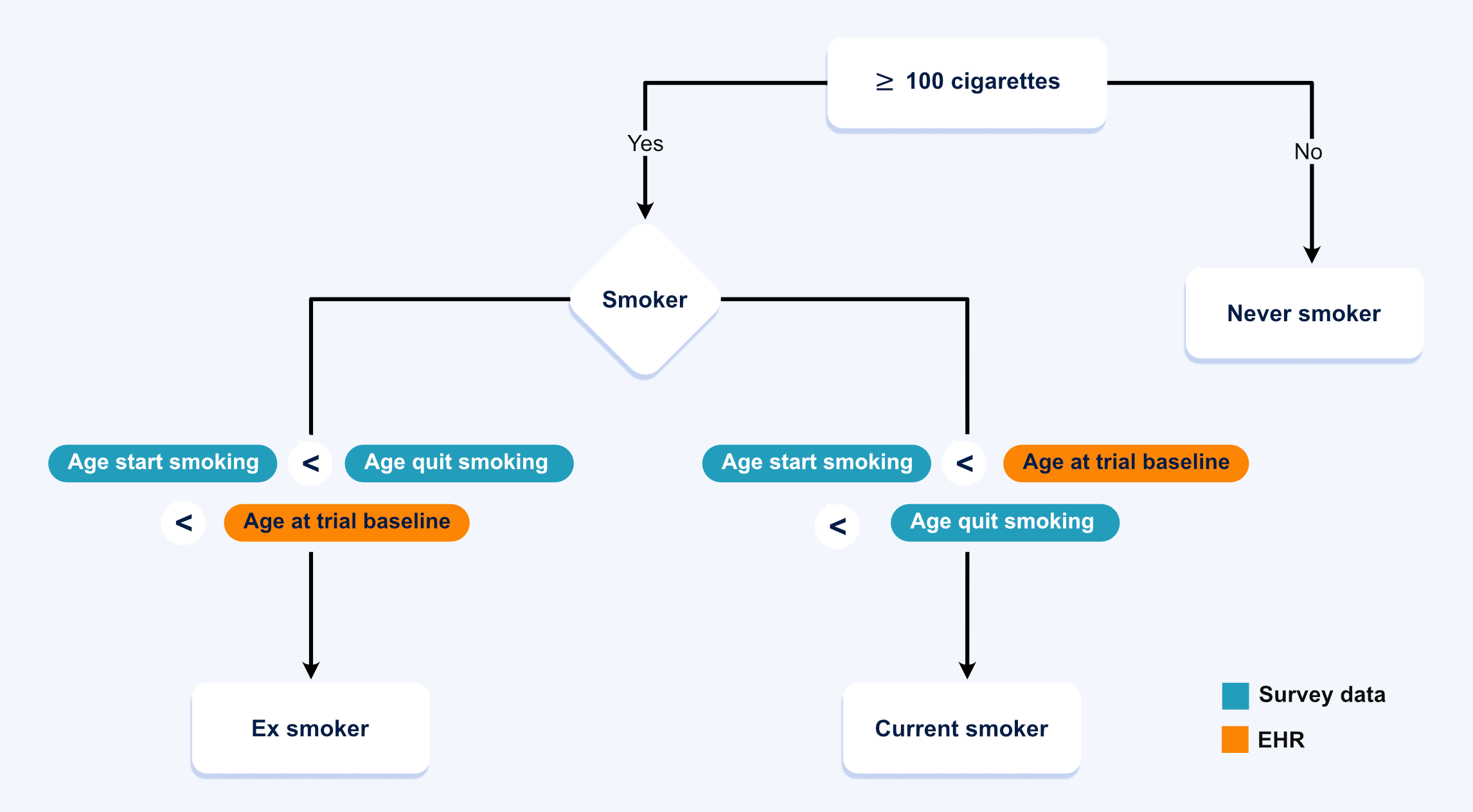


**Figure S1.** Diagram illustrating the algorithm used to determine smoking status at or before the trial baseline.

# Simplified directed acyclic graph


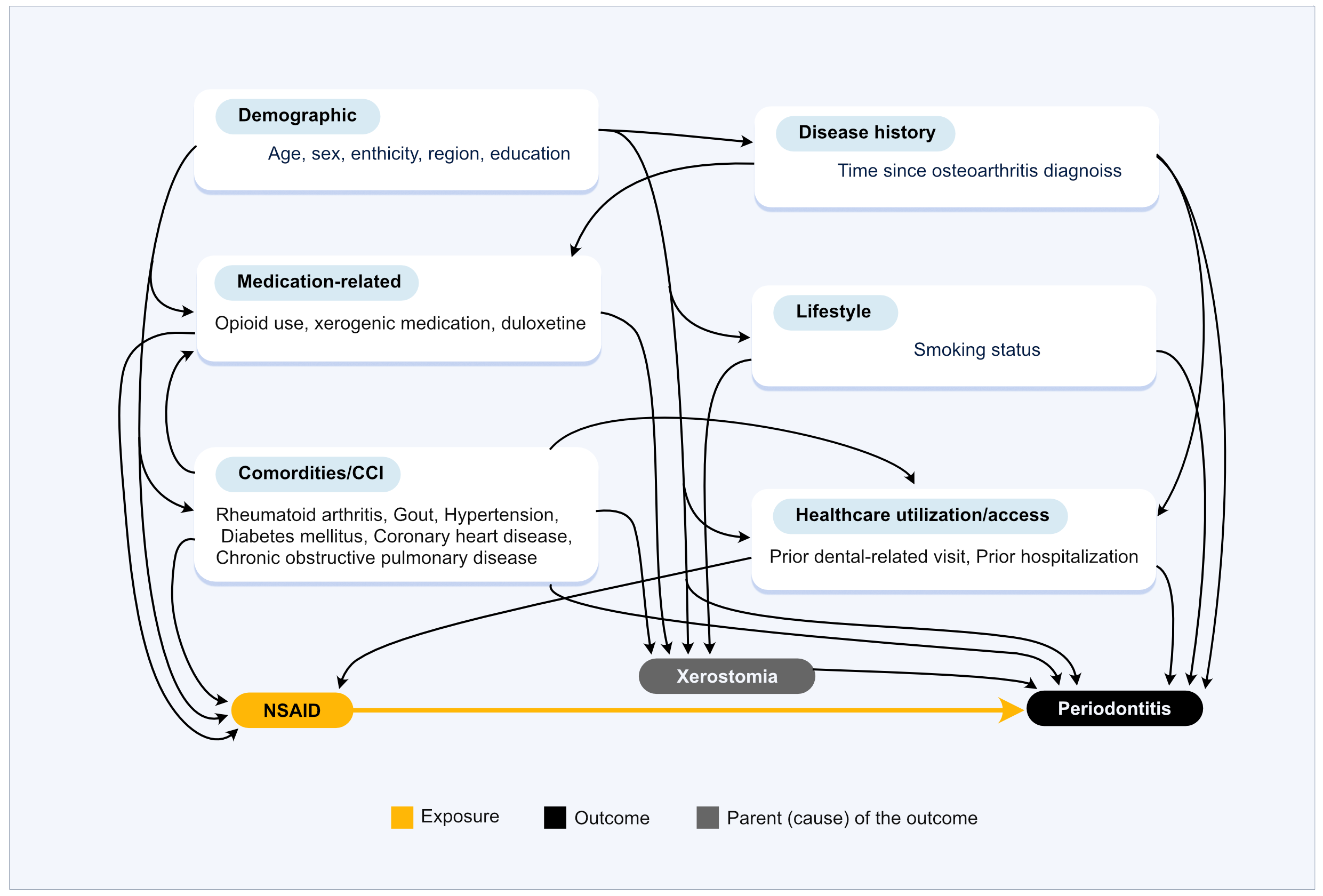


**Figure S2.** Directed acyclic graph (DAG) to identify confounders of the focal relationship between NSAID initiation and periodontitis. *NSAID: non-steroidal anti-inflammatory drugs, CCI: Charlson comorbidity index.* **Note**: For visual clarity, directed edges between and within individual covariate are omitted in the simplified DAG.

# Subgroup analysis

## NSAID affinity

To examine potential differences by NSAID type, we categorized agents based on their COX inhibition profiles (IC₅₀ values), distinguishing non-selective inhibitors from those with predominant COX-2 affinity (hereafter referred to as preferential COX-2 inhibitors) (Pawson et al., 2014). In borderline cases, classification was additionally done considering gastrointestinal toxicity. Marketing status was checked using the Orange Book FDA (U.S. Food and Drug Administration, 2024).

**Table S5.** Classification of NSAIDs based on COX affinity.

| **Drug** | **NSAID classification** | **Marketing status** | **Number users (%)** |
| --- | --- | --- | --- |
| Celecoxib | Preferential COX-2 (coxib) | Prescription | 700 (6.54) |
| Diclofenac^1^ | Non-selective | Prescription | 649 (6.07) |
| Diflunisal | Non-selective | Prescription | 27 (0.25) |
| Etodolac | Preferential COX-2 (higher affinity) | Prescription | 286 (2.67) |
| Fenoprofen | Non-selective | Prescription | ** |
| Flurbiprofen | Non-selective | Prescription | ** |
| Ibuprofen | Non-selective | OTC low strength  Prescription | 4035 (37.71) |
| Indomethacin | Non-selective | Prescription | 390 (3.64) |
| Ketoprofen | Non-selective | Prescription | 34 (0.32) |
| Ketorolac | Non-selective | Prescription | 156 (1.46) |
| Meloxicam | Preferential COX-2 (higher affinity) | Prescription | 985 (9.21) |
| Nabumetone | Non-selective | Prescription | 254 (2.37) |
| Naproxen | Non-selective | OTC low strength  Prescription | 2540 (23.74) |
| Oxaprozin | Non-selective | Prescription | 73 (0.68) |
| Piroxicam | Non-selective | Prescription | 138 (1.29) |
| Rofecoxib | Preferential COX-2 (coxib) | Prescription | 159 (1.49) |
| Salsalate | Non-selective | Prescription | 137 (1.28) |
| Sulindac | Non-selective | Prescription | 103 (0.96) |
| Tolmetin | Non-selective | Prescription | ** |
| Valdecoxib | Preferential COX-2 (coxib) | Prescription | 22 (0.21) |

*Cells with fewer than 20 participants were replaced by **, in accordance with the All of Us Research Program and Statistics Dissemination Policy. ^1^Even though diclofenac has a relatively high affinity for COX-2, more than the other non-selective NSAIDS, it has a substantial gastrointestinal risk.*

## Sex, ethnicity, and education


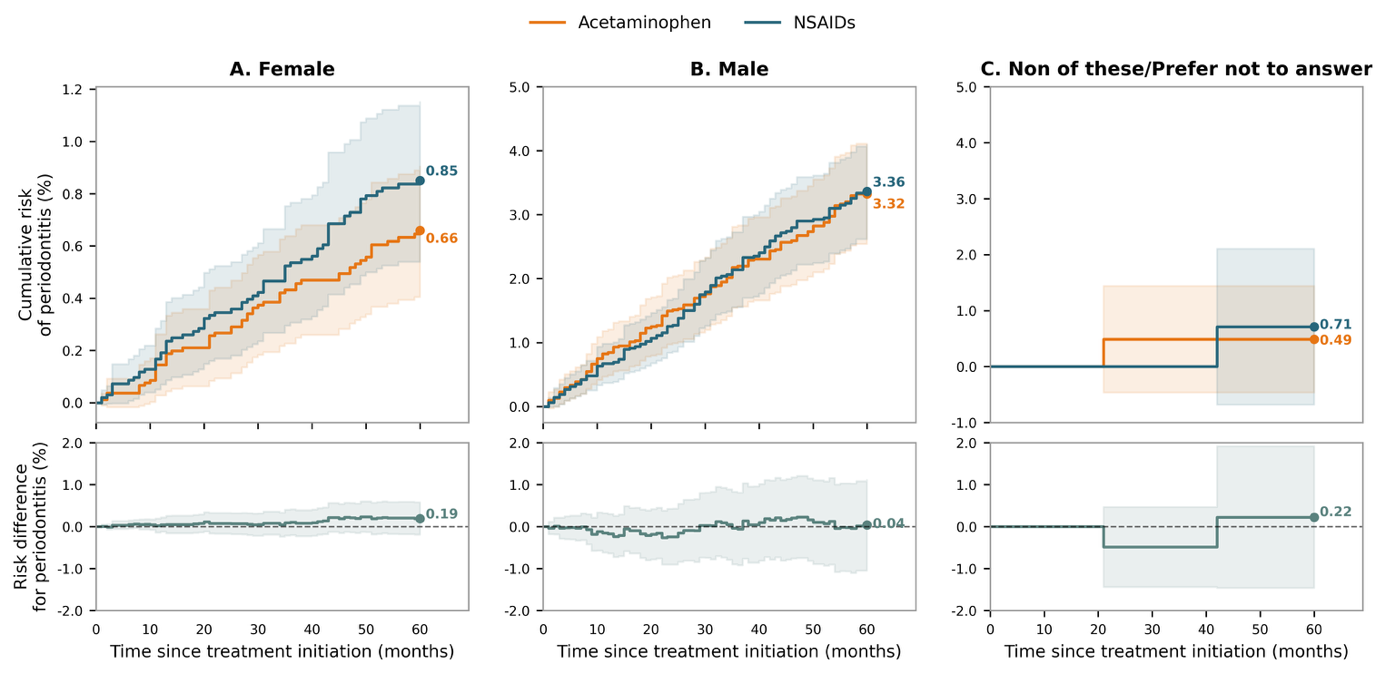


**Figure S3.** Cumulative risk and risk difference by sex among individuals with osteoarthritis initiating NSAIDs or acetaminophen. The top panels display the cumulative risk over time for NSAID initiators (blue lines) and acetaminophen initiators (orange lines). The bottom panels show the estimated risk differences, with the dashed gray line indicating the null effect. Shaded areas represent 95% confidence intervals.


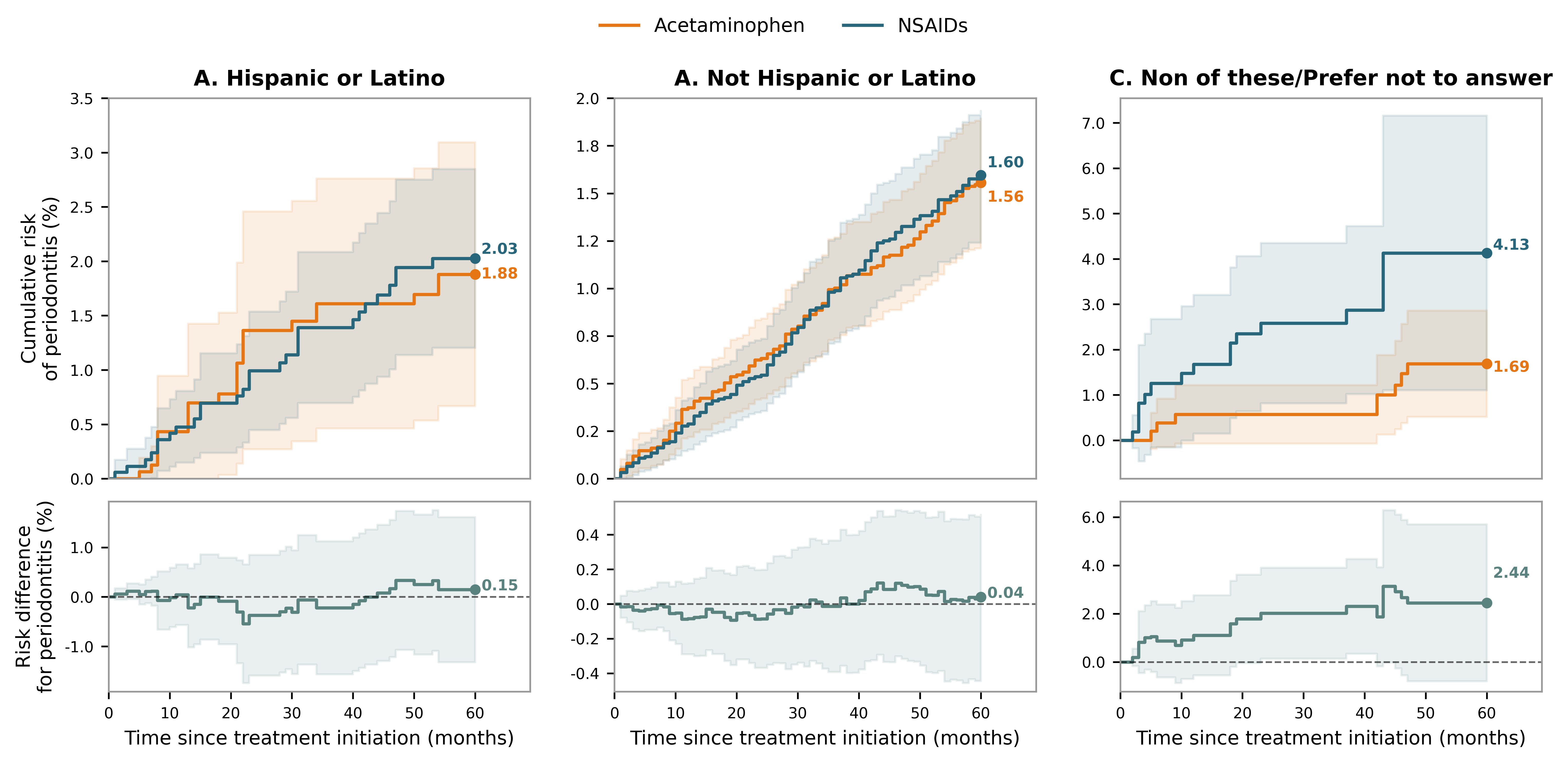


**Figure S4.** Cumulative risk and risk difference by ethnicity among individuals with osteoarthritis initiating NSAIDs or acetaminophen. The top panels display the cumulative risk over time for NSAID initiators (blue lines) and acetaminophen initiators (orange lines). The bottom panels show the estimated risk differences, with the dashed gray line indicating the null effect. Shaded areas represent 95% confidence intervals.


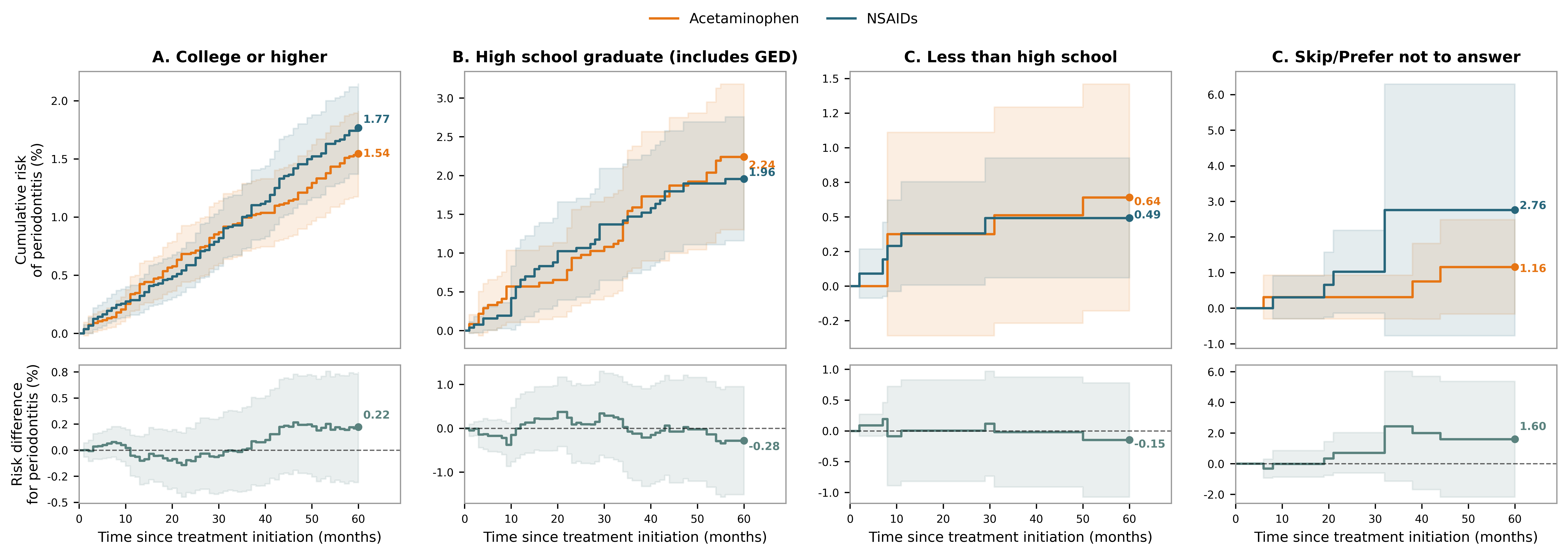


**Figure S5.** Cumulative risk and risk difference by education level among individuals with osteoarthritis initiating NSAIDs or acetaminophen. The top panels display the cumulative risk over time for NSAID initiators (blue lines) and acetaminophen initiators (orange lines). The bottom panels show the estimated risk differences, with the dashed gray line indicating the null effect. Shaded areas represent 95% confidence intervals.

# Negative control outcome

The negative control outcome (NCO) analysis used the OMOP concept ID 133228 to identify dental caries. Individuals with a prior diagnosis of dental caries were excluded to ensure that the outcome reflected incident, rather than prevalent, cases.

# Additional results

## Propensity score evaluation


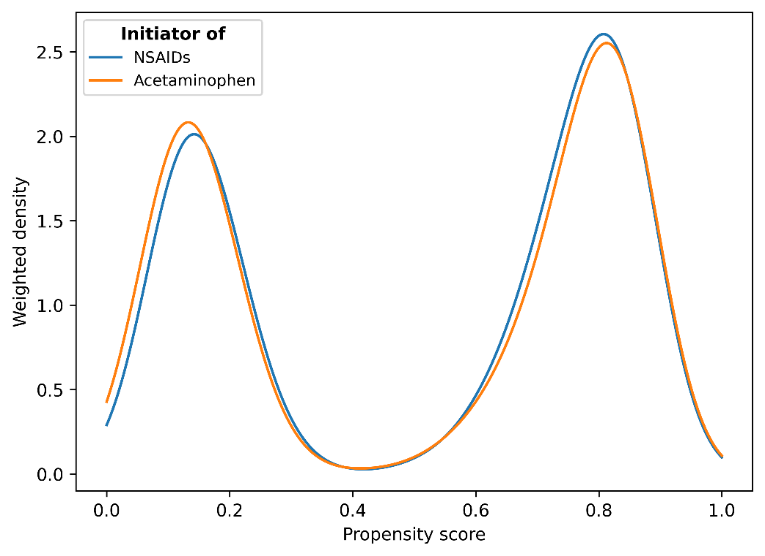


**Figure S6.** Overlap of the weighted propensity score between patients initiating NSAIDs and those initiating acetaminophen.


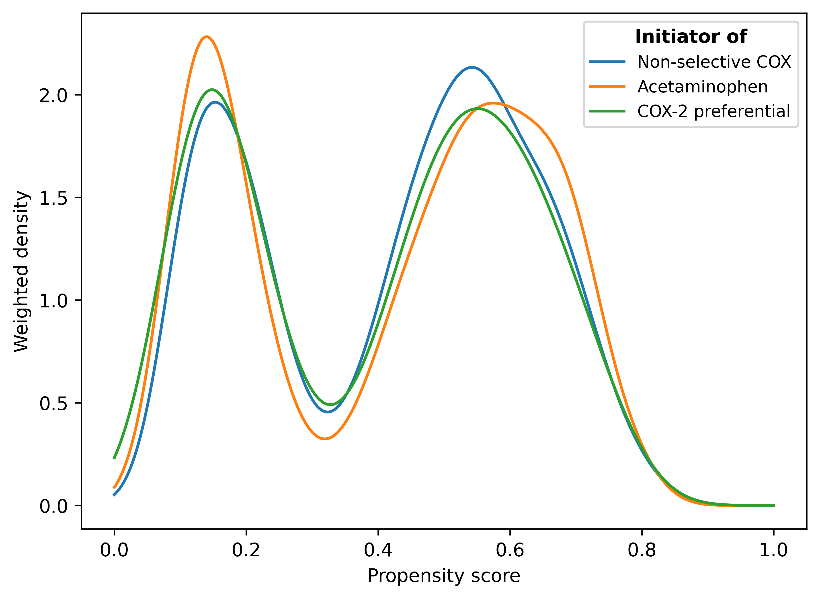


**Figure S7.** Overlap of the weighted propensity score among patients initiating COX-2 preferential agents, non-selective COX NSAIDs, and acetaminophen.

**References**

Fortin, S. P., Reps, J., & Ryan, P. (2022). Adaptation and validation of a coding algorithm for the Charlson Comorbidity Index in administrative claims data using the SNOMED CT standardized vocabulary. *BMC Medical Informatics and Decision Making*, *22*(1). https://doi.org/10.1186/s12911-022-02006-1

Patel, N., Miller, D. P., Snavely, A. C., Bellinger, C., Foley, K. L., Case, D., McDonald, M. L., Masmoudi, Y. R., & Dharod, A. (2020). A Comparison of Smoking History in the Electronic Health Record With Self-Report. *American Journal of Preventive Medicine*, *58*(4), 591–595. https://doi.org/10.1016/j.amepre.2019.10.020

Pawson, A. J., Sharman, J. L., Benson, H. E., Faccenda, E., Alexander, S. P. H., Buneman, O. P., Davenport, A. P., McGrath, J. C., Peters, J. A., Southan, C., Spedding, M., Yu, W., Harmar, A. J., & NC-IUPHAR. (2014). The IUPHAR/BPS Guide to PHARMACOLOGY: An expert-driven knowledgebase of drug targets and their ligands. *Nucleic Acids Research*, *42*(Database issue), D1098-1106. https://doi.org/10.1093/nar/gkt1143

Polubriaginof, F., Salmasian, H., Albert, D. A., & Vawdrey, D. K. (2017). Challenges with Collecting Smoking Status in Electronic Health Records. *AMIA ... Annual Symposium Proceedings. AMIA Symposium*, *2017*, 1392–1400.

U.S. Food and Drug Administration. (2024). Approved Drug Products with Therapeutic Equivalence Evaluations (Orange Book), 44th ed. U.S. Food and Drug Administration. https://www.accessdata.fda.gov/scripts/cder/ob/
